# Using relevance mapping methodology to design an adolescent mental health intervention in India

**DOI:** 10.1080/16549716.2020.1775062

**Published:** 2020-06-26

**Authors:** Maya M. Boustani, Eric Daleiden, Adam Bernstein, Daniel Michelson, Resham Gellatly, Kanika Malik, Vikram Patel, Bruce Chorpita

**Affiliations:** a Department of Psychology, Loma Linda University, Loma Linda, CA, USA; b PracticeWise, Satellite Beach, FL, USA; c School of Psychology, University of Sussex, Brighton, UK; d Department of Psychology, University of California, Los Angeles, CA, USA; e Sangath, Goa, India; f Department of Global Health and Population, Harvard Medical School, Cambridge, MA, USA

**Keywords:** Global mental health, transdiagnostic, adolescence

## Abstract

**Background:**

Adolescents in low and middle-income countries experience pronounced mental health needs in contexts where infrastructure and resources are scarce. While evidence-based treatment are readily available, they may not fit the unique needs of certain contexts.

**Objective:**

This manuscript illustrates the systematic process of applying ‘relevance mapping’ methodology to leverage the youth mental health evidence base to identify candidate practices for inclusion in the development of a contextually appropriate psychological treatment protocol for common adolescent mental health problems in India.

**Methods:**

The practice identification was informed by two datasets obtained from adolescent samples in India. The first was an epidemiological dataset from a large community sample in Goa (N = 2,048); the second incorporated ‘youth top problems’ reported by service-seeking students presenting to school counsellors in Goa and Delhi (N = 78). Problems identified in each dataset were categorized using structured codes. Problem codes and youth demographics were then indexed against a database of hundreds of evidence-based psychological treatments and their associated clinical trials. This methodology revealed the most common practice elements (discrete therapeutic strategies) and their most efficient combinations with evidence of effectiveness matching the demographics and diagnostic category (anxiety, disruptive behaviors and depression) prevalent in the planned treatment population.

**Results:**

For anxiety, the most common practice elements for this age group were exposure, cognitive coping, and psychoeducation. For disruptive behaviors, the most common practices were problem-solving, goal-setting, and rapport-building. For depression, cognitive coping, behavioral activation, and psychoeducation were the most common practice elements.

**Conclusion:**

These practice elements provided the treatment development team with a preliminary list of candidate content for the development of an intensive psychological treatment within a stepped care service model to address common adolescent mental health problems in schools in India.

## Background

Low- and middle-income countries (LMICs) experience pronounced mental health burdens, where needs are vast, resources are scarce, and stigma is high [1]. This is compounded by the scarcity of context-specific intervention research, with most evidence-based treatments (EBTs) evaluated in high-income countries, under conditions that include robust infrastructure and delivery by trained workforces with access to expert supervision [2]. As such, there are concerns about the applicability, scalability, and sustainability of EBTs transported into low-resource settings, along with the acceptability of treatments developed in western countries when applied in contexts with different cultural beliefs around mental health [3].

Calls for robust interventions for low-resource settings have led to a focus on designing interventions in public health contexts [4]. This approach differs from the strategy of selecting and adapting a single existing EBT and instead assembles the intervention from available components, jointly considering the local context and relevant evidence base [5,6]. Such an approach is compelling when the target problems in the population might otherwise require multiple standalone EBTs. Instead, building a system from components allows for a coordination of all relevant practices into a single organized system, which can have advantages in terms of efficiency, scalability, and exception handling (e.g., changing clinical focus to address interference or comorbidity).

Building an intervention in context necessitates important design considerations [7] such as [1] accounting for the local epidemiology [2] addressing the majority of targeted problems with minimum cost and effort [3] reflecting on challenges that affect implementation and utilization (e.g., engagement barriers, comorbidity, stigma, low parental involvement, poor literacy, stressors); and [4] understanding workforce characteristics such as local capacity, training burden, and provider preferences [8,9].

A key consideration for intervention designers taking this approach is to identify which practices to include, considering the characteristics of the target population (e.g., what practices fit the clinical problems and demographics), as well as the characteristics of the local workforce (i.e., what practices are acceptable, scalable, and sustainable with the intended providers). This paper describes the practice selection component of a larger treatment development process. Specifically, we relied on relevance mapping methodology [10] to identify practice elements (e.g., discrete therapeutic procedures such as relaxation or problem-solving) derived from a database of EBT protocols [11], indexed against the most common mental health concerns found in two Indian reference samples. The purpose was to identify candidate practice elements of direct relevance to common mental health problems among school-going adolescents in India, and thus to consider their inclusion in a new treatment that would meet their specific needs. These practice elements have been derived from over 1,000 randomized controlled trials from the literature, encompassing a number of treatment families, including cognitive-behavior therapies and others [12,13].

Relevance mapping is a methodology that allows for direct and systematic comparison of client characteristics in a service sample to participant characteristics in a research study [10,13–15]. This methodology was applied as part of ‘PRemIum for aDoleEscents’ (PRIDE), a 5-year research program aimed at developing and evaluating a transdiagnostic, stepped care, service model for common mental health problems among school-going adolescents in India. ‘Step 1’ is a brief, low-intensity intervention focused on teaching adolescents problem-solving skills to cope with daily stressors [16]. ‘Step 2’ is a more intensive treatment focused on persistent internalizing and externalizing difficulties that failed to remit after Step 1 (see Chorpita et al., in press [17] for a full description, including how the results from this study informed the development of the intervention). The PRIDE program was intended to serve school-going adolescents [18,19] in Delhi and Goa, India, a context in which anxiety, depression, and conduct problems account for the majority of mental health concerns [20], and in which there is a scarcity of trained professionals to meet the considerable mental health burden [21]. Thus, this study is intended to illustrate how the evidence-base for youth mental health was leveraged to inform practice selection as one part of designing a new intervention in context.

## Methods

The identification of candidate practice elements was based on two types of analyses [1]: relevance mapping of an epidemiological dataset [22]; and [2] a descriptive analysis of Youth Top Problems [23] data from local service-seeking youth.

The epidemiological data originated from a previously approved study. Approvals for PRIDE formative research activities (data from local youth in Goa and Delhi) were obtained from the Indian Council of Medical Research and the Institutional Review Boards of the sponsor (Harvard Medical School); a collaborating academic partner (London School of Hygiene and Tropical Medicine); and the two implementing organizations in India (Public Health Foundation of India and Sangath). Informed consent was collected from all adolescents aged 18 years or older, with informed assent and corresponding parental consent obtained for minor adolescents.

### Measures

#### Epidemiological reference sample: development and well-being assessment (DAWBA) and structured clinical interview

Pillai et al. (2008) used the DAWBA [24] (parent report) with both the parent and the adolescent to assess for psychopathology and provide a DSM-IV diagnosis when appropriate.

#### Service reference sample: youth top problems

The Youth Top Problems (YTP) is an idiographic measure of self-reported problem severity [23] and was completed by all participants. Respondents nominate up to three problems they are most concerned about. Each problem is rated on a scale from 0 to 10 (with 10 indicating highest severity/concern). Finally, respondents rank-order the problems in order of priority. Problems identified using this method match those reported via standardized measures (such as the Child Behavior Checklist [25]), while adding specificity that is clinically relevant and useful for treatment planning [23].

### Procedures

In order to identify candidate practice elements, we used two procedures. The first was a relevance mapping analysis examining practice elements that matched individual youth from the epidemiological dataset on mental health problems, age, and gender to sample characteristics in a study dataset [10].

The second procedure was coding of the YTP data reported by the service-seeking sample, followed by a search for the most common elements of treatments corresponding to the age range and most prevalent problems reported on the YTP. Both procedures relied on the PracticeWise Evidence Based Services database (PWEBS; https://www.practicewise.com) to provide information about EBTs for adolescents and associated practice elements.

By organizing the literature in this way, one can identify the most commonly used strategies found in EBTs that are suitable to address specific problems for a particular population. Although the majority of EBTs that have been tested in published research have been studied in high-resourced countries, a body of studies testing treatments for youth in LMICs has emerged (n = 46 papers), with four taking place in India. In order to leverage a larger literature, we did not include context, ethnicity or geographic location as filters in our search. We included only age, gender, and problem.

#### Relevance mapping and relevant practices analysis of epidemiological dataset

The epidemiological dataset was analyzed using relevance mapping [10,14,15]. This procedure examines the clinical (problem area) and demographic (e.g., age, gender) profiles of individuals in a given population and determines which published trials included participants with matching characteristics and whose results supported evidence for the effectiveness of the treatment in question. Relevance mapping shows which practice elements are applicable to the most youth, and what are the smallest sets of practices sufficient to constitute treatments from the study dataset that maximize the percentage of youth ‘covered’ (i.e., for whom there was a matching EBP in the study dataset). We cross-referenced the epidemiological sample from Goa against psychological treatment protocols indexed in the PWEBS database (PracticeWise, 2019) to determine the proportion of adolescents that would potentially be ‘covered’ by existing EBTs (i.e., the proportion of adolescents whose characteristics met the corresponding inclusion criteria for the trials in which the EBTs were tested and found to be effective). We considered three scenarios to determine ‘coverage’: we required adolescents to match research participants on either (a) problem, (b) problem and age, (c) problem, age, and gender. For the purposes of this investigation, all DSM–IV diagnoses from the DAWBA were grouped to 10 broad ‘problem’ categories (the mapping of all diagnoses to problem areas is available upon request). For scenarios involving problem, the relevant literature was limited to studies in which all participants were ages 12–19 years. In each of these scenarios, adolescents covered by at least one trial (i.e., at least one trial tested an intervention that included participants that matched our sample on their problem, problem and age, or problem, age, and gender) from among all those in the PWEBS database were deemed ‘coverable.’ Additionally, the list of matches between adolescents and trials was the starting point for a ‘relevant practices’ analysis, in which we identified relevant practice elements from those interventions to find the smallest sets of practice elements that together would cover a maximum number of adolescents.

#### YTP coding and common practices analysis of service-seeking dataset

YTP responses were translated into English by a bilingual research assistant and entered into a spreadsheet with qualitative descriptions of the self-identified problem, severity rating and ranking. Each entry was coded by a co-author (RG) and double-coded by the first author (MB) using the high-level categories: anxiety, attention problems, depression, disruptive behavior, trauma and substance use [26]. Self-identified problems that fell outside of these clinical areas were coded according to the following high-level codes: socio-emotional health (e.g., family functioning); academic (e.g., school attitudes); and social (e.g., low self-esteem). Discrepancies in the coding were resolved via discussion. Subsequently, we conducted a search on the PWEBS database filtering to identify practice elements present in EBTs for the most common problems, for the age group of interest. We decided to focus only on studies in which *all* participants were between the ages of 12 and 19 years (the age range from our service-seeking sample) to maximize generalizability of our findings to our target population. In order to identify interventions that were tested with a high level of research rigor, we restricted our search to interventions with the strongest amount of evidence to support their effectiveness. For this study, interventions had to have at least two randomized trials demonstrating efficacy, be manualized, and their effectiveness demonstrated by at least two investigator teams (see https://www.practicewise.com/Community/BlueMenu). All results reported below reflect findings from interventions with this level of rigor and will be referred to as ‘evidence-based’.

## Results

Participants in the epidemiological reference sample [22] were *N* = 2,048 adolescents (*n* = 1,031 male; *n* = 1,017 female) aged 12–16 years (*M* = 13.8 years), drawn from a community sample in Goa, India with data collected in two waves [22]. From this community sample, 37 youth (1.8%) during the first wave, and 54 youth (2.6%) across the two waves presented with a DSM-IV diagnosis. Our analyses are based on this sub-sample of 54 youth presenting with psychopathology during either wave of the study. Anxiety was the most common diagnosis (53.7%, n = 29), followed by depression (29.6%, n = 16), behavior disorders (7.4%, n = 4), attention deficit hyperactive disorder (10.8%, n = 5), and other disorder (13%, n = 7).

The service reference sample was comprised of 78 adolescents (*n* = 48 male; *n* = 30 female) aged 12–19 years (*M* = 15.17 years) who were seeking psychological help from counsellors at three government schools in Delhi and six government aided schools in Goa [16].

By using data from both an epidemiological sample and a service-seeking sample, we sought to understand the common mental health problems in the community overall, and within a clinical sample in India. See Table 1 for additional details about these samples.

**Table 1.** Sample details.

	Epidemiological Reference Sample (Pillail et al., 2008) N = 2048	Service Seeking Sample (Michelson et al., 2019*) N = 78 (Delhi n = 48; Goa n = 30)		
Gender	1031 males; 1017 females	48 males; 30 females		
Age	12 to 16 (M = 13.8)	12 to 19 (M = 15.17)		
Prevalence of psychological problems*	N = 54 (2.6%)	BOTH SITES** N = 65; 83%	DELHI N = 38; 79%	GOA N = 27; 90%
Anxiety	1%	30%	29%	30%
Depression	0.5%	24%	29%	15%
Disruptive	0.4%	24%	24%	30%
ADHD	0.2%	18%	18%	19%
Substance	0%	1.5%	0%	4%
Trauma	0%	1.5%	0%	4%
	* based on Development & Well-Being Assessment	*based on problem ranked #1 on YTP **part of the sample (n = 13; 18%) reported non-clinical problems		
Setting	One urban and one rural area in Goa (community).	Three Government-run schools (1 all-girls and 2 all-boys schools) in Delhi (n = 48) and Six Government-aided schools (all co-educational) in Goa (n = 30).		
Sampling	Communities were randomly selected out of six urban wards and four rural communities between October 2002 and May 2003. Youth were identified from family registers and door-to-door surveys.	Schools were selected in consultation with the local school authorities, based on perceived need for counselling services. Youth were primarily self-referred (Delhi) and teacher-referred (Goa).		
Data Collection	Face-to-face interviews were conducted by trained researchers using a Konaki translation of the Development and Well-Being Assessment to diagnose DSM-IV disorders	Students completed the idiographic Youth Top Problems (YTP) measure, which was administered by trained counsellors. The YTP asks the respondent to identify up to three main psychosocial concerns, defined in their own words. Each of the nominated concerns is then scored from 0 (not a problem) to 10 (huge problem). The respondent also ranks these problem descriptions in order of priority (”which is the most important problem for you right now”).		

* The YTP sample was partly made up from a Delhi-based subsample used in a previously published study (Michelson et al., 2019) and partly from a cohort in Goa that has not been reported elsewhere.

### Overall review of findings

Both sets of analyses (relevance mapping of the epidemiological dataset and common practices analysis of the service-seeking dataset) indicated that adolescents were most likely to report concerns related to anxiety, depression, and disruptive problems. Findings specific to each of the analyses are outlined below.

### Relevance mapping and relevant practices analysis of epidemiological dataset

The relevance mapping population coverage analysis yielded similar results across the problem only, problem and age, and problem, age, and gender scenarios. As such, we report our results for the problem and age scenario. Specifically, 76% of adolescents from the epidemiological sample were covered by at least one EBT, meaning that at least one treatment targeting at least one problem could be identified for 41 of the 54 adolescents with a DSM-IV diagnoses in our epidemiological sample. No relevant EBTs were identified for adolescents with diagnoses in the ADHD or other problem categories.

The relevance mapping practice minimization analysis yielded slight differences between the problem only scenario and the scenarios which also included age as a matching factor (i.e., the problem and age, and problem, age, and gender scenarios). The smallest set of treatments needed to cover adolescents in the problem only scenario included the following twelve practice elements (alphabetically): exposure, family therapy, praise, psychoeducation for caregiver, tangible rewards, therapist praise/rewards, and cognitive or psychoeducation for child. The models matching on (a) problem area and age, and (b) problem area, age, and gender yielded the same solution, which covered 61% of adolescents. The smallest set of treatments needed to cover these adolescents required all of the ones from the problem only solution, plus four additional practice elements (alphabetically): activity selection, cognitive, goal-setting, and problem-solving.

### YTP coding and analysis of common practices analysis of service-seeking dataset

A total of 175 problems were coded from 78 participants. Of these problems, 35 fell into a non-symptomatic category: socio-emotional health (*n* = 16; e.g., family functioning or peer/sibling conflict); social (*n* = 11; e.g., low self-esteem); and academic (*n* = 8; e.g., poor performance). The remaining 140 problems were coded into the following categories: anxiety (29%), disruptive problems (21%), depression (19%), attention problems (19%), trauma (2%), and substance use (1%). This distribution of problems in this sample is similar to the epidemiological dataset. The 64 symptomatic problems that were ranked as top priority by the adolescents fell into the following categories: anxiety (30%), disruptive problems (27%), depression (23%), attention problems (19%), and trauma (2%). Based on the YTP coding, the protocol development team decided that the intervention should focus on the most common adolescent mental health problems in this context for which evidence-based psychosocial treatment procedures are available. Thus, although adolescents frequently noted attention problems, we did not consider including any psychosocial procedures to address them, because our PWEBS search did not reveal any evidence-based procedures for ADHD in this age range. Further, we did not include trauma and substance use, due to their very low prevalence. Next, we conducted PWEBS searches to identify the most common practice elements from EBTs that addressed the top three most prevalent problem areas for adolescents aged 12 to 19 years: anxiety, disruptive problems, and depression. A total of 51 elements mapped onto one of the identified problem areas. Due to the school-based nature of our planned intervention context, we decided to only include elements that do not require parent participation (n = 34). Table 2 highlights the definitions of these elements and Table 3 provides a breakdown of the top practice elements for this age group and these problem areas. When considering practice elements corresponding to all three target areas, the ten most widely utilized elements were (mean prevalence): cognitive coping (67% of study groups); problem-solving (47%), psychoeducation with youth (44%), maintenance (41%), social skills training (39%), self-monitoring (38%), goal-setting (36%), relaxation (28%), therapist praise (24%), communication skills (23%), self-reward (23%), and relationship building (23%).

**Table 2.** Practice elements definitions abbreviated from PracticeWise (2009).

Practice elements	Definition
Activity Selection	Participation in positive activities to improve mood
Assertiveness Training	Exercises designed to promote the youth’s ability to assert their needs appropriately with others
Cognitive Coping	Any techniques designed to alter interpretations of events through examinations of the youth’s reported thoughts
Communication Skills	Training for youth in how to communicate more effectively with others
Exposure	Direct or imagined experience with a stimulus with the goal of desensitization
Functional Analysis	The study of antecedents and consequences that impact a youth’s behavior
Goal-setting	Selection of therapeutic goals, including measurement of success in achieving those goals
Guided Imagery	Visualization or guided imagination to rehearse successful performance
Maintenance	Exercises and training designed to consolidate skills already developed to minimize the chance that gains will be lost in the future
Modeling	Demonstrations to the youth of a desired behavior
Peer Pairing	Pairing with another youth to allow for reciprocal learning or skills practice
Problem Solving	Training in the use of techniques, discussions, or activities designed to bring about solutions to targeted problems
Psychoed Child	The formal (usually didactic) review of information with youth
Relationship Building	Strategies to improve the relationship between youth and therapist
Relaxation	Techniques or exercises designed to induce physiological calming
Self-monitoring	Repeated measurement by youth of a target index
Self-reward	Techniques to encourage youth to self-administer rewards when performing desired behaviors
Social Skills Training	Providing constructive information, training, and feedback to improve interpersonal verbal or non-verbal functioning
Therapist Praise	The administration of praise by the therapist to promote a desired behavior in youth

**Table 3.** Top practice elements (%) present in evidence-based interventions for 12 To 19 years-old for each diagnostic category (Practices requiring parental participation excluded).

	Across PBM areas (N = 75 papers)	Anxiety (n = 27 papers)	Depression (n = 23 papers)	Disruptive (n = 25 papers)
Cognitive coping	67	67	83	36
Problem-solving	47	30	52	60
Psychoeducation with youth	44	52	65	16^a^
Maintenance	41	26	52	44
Social skills training	39	26	39	52
Self-monitoring	38	41	48	24^a^
Goal-setting	36	7^a^	52	48
Relaxation	28	41	35	8^a^
Therapist praise	24	15	26^a^	32
Communication	23	4^a^	35	32
Self-reward	23	19	39	12^a^
Relationship building	23	7^a^	17^a^	44
Exposure	-^b^	93	0^a^	0^a^
Activity selection	-^b^	0^a^	70	0^a^
Modeling	22^a^	7^a^	22^a^	36
Guided imagery	15^a^	4^a^	35	8^a^
Assertiveness	20^a^	7^a^	22^a^	32
Functional analysis	-^b^	0^a^	4^a^	32
Peer pairing	-^b^	15	0^a^	8^a^

^a^not in the top 10 for this target area; ^b^ not in all target areas

All results reflect findings from peer-reviewed research journal articles describing a randomized clinical trial that tested an evidence-based intervention with a study sample that included 12–19-year-olds. *Anxiety* was the most commonly coded problem on the YTP (*n* = 41), and was the focus of interventions in 27 articles. The most common practice elements were exposure (93%), cognitive coping (67%), psychoeducation with youth (52%), self-monitoring (41%), relaxation (41%), problem-solving (30%), maintenance/relapse prevention (26%), social skills training (26%), self-reward (19%), peer pairing (15%), and therapist praise (15%).

*Disruptive behaviors* were coded 30 times on the YTP and were the focus of interventions in 25 articles. The most common practice elements were: problem-solving (60%), social skills training (52%), goal-setting (48%), maintenance/relapse prevention (44%), rapport-building (44%), cognitive coping (36%), modeling (36%), assertiveness training (32%), communication skills (32%), and functional analysis (32%).

*Depression* was coded 27 times on the YTP and was the focus of 23 articles. The most common practice elements were: cognitive coping (83%), activity selection (70%), psychoeducation with youth (65%), goal-setting (52%), maintenance/relapse prevention (52%), problem-solving (52%), self-monitoring (48%), self-reward (39%), social skills training (39%), communication skills (35%), guided imagery (35%), and relaxation (44%).

## Discussion

The goal of this effort was to develop a parsimonious psychological treatment protocol that could address the majority of mental health problems presented by adolescents in Indian schools. We used relevance mapping methodology [10] to align the epidemiology from 132 youth in India with the evidence base from a database of over 1,000 research papers on youth mental health interventions (PWEBS; https://www.practicewise.com) to generate a list of candidate practice elements for inclusion in the protocol. The work presented here represents just one of several phases involved in the development of this protocol. Findings from both the epidemiological and the service-seeking samples indicated that adolescents in this context are most likely to exhibit anxiety, disruptive behaviors and depression. These problems were similar across both samples, and similar to the prevalence of adolescent psychopathology globally [27]. As such, we believe our results are generalizable to other parts of the world. However, in the YTP data, these globally common mental health concerns were often framed as contextual risks and idioms. Given that self-defined problems may drive initial help-seeking, we consider an understanding of contextualized psychosocial difficulties key to effective intervention design. For instance, youth stated ‘*students tease me and I feel sad and like I want to cry*’ (depression); ‘*when other students disturb me and I am not able to concentrate, I feel like beating them*’ (disruptive behavior); ‘*my mother is not keeping well, I keep thinking about her and what will happen to her – I am worried about her health*’ (anxiety). Accordingly, we considered both the relevant practice content indicated by the analysis and the need to organize practices into a stepped care approach, such that the first line intervention addresses undifferentiated or transient distress linked to common difficulties (Step 1 [16],), followed by a more intensive intervention for more overt psychopathology (Step 2 [17],).

This study demonstrates that, even when developing a new intervention for a new context, we do not need to ‘reinvent the wheel’. The parts of the intervention (practice elements) tested in high income countries were relevant for youth in this context. Based on our analyses from the global evidence, we generated a list of 19 candidate practices for adolescents (see Tables 2 and 3) for possible inclusion in the intervention. These practice elements overlapped with some practices elements found in a recent review of life skills programming in LMICs [28] such as communication skills, problem-solving, and exposure. Our list of candidate practice elements allowed the treatment development team to make a selection and combine the elements into a context-specific protocol to address the three most common mental health problems among Indian adolescents. A number of downstream decisions were made once the list of practice elements was generated. Although it provided a starting point for the treatment development team, they did not simply take all the practice elements, nor did they prioritize them merely based on how common they are in the literature. In order to determine which practice element should be included in the intervention, the team discussed issues related to parsimony, fit with local context, scalability, generalizability, feasibility, acceptability of strategies by both local providers and adolescents, and sustainability beyond the duration of the research project. The team also considered local constraints such as limited time, shortage of a trained workforce, and lack of infrastructure. In close collaboration with local experts (school counselors), the team discussed several options that could address these constraints. For instance, they discussed selecting practices that are familiar and acceptable to the local culture, such as relaxation. They also considered balancing the selection of practices that are transdiagnostic (e.g., problem-solving which was common across the three problem areas) and practices that are focal to a specific problem area (e.g., exposure for anxiety). In addition, the team explored how different practice elements would flow with each other to determine if they can all work within a single treatment protocol, and how the protocol would flow at the system level, both within the larger stepped care service model and within the entire array of services in India. Indeed, youth are referred to this treatment after completing an intervention that is the lower step in a stepped care model [16]. Thus, this protocol had to be compatible with the lower step, and it had to build on its content. Although our findings may be generalizable to other parts of the world, this information might best be viewed as a starting point for use by each community to develop a unique protocol based on local needs and resources, as outlined above. The full details about the intervention development, including the final selection of practice elements are available elsewhere [17].

### Limitations

There are a number of limitations to consider when interpreting the findings from this study. First, a large majority of the studies that we consulted were conducted in Western countries, potentially limiting their relevance to India. Nevertheless, we hope that our efforts in considering the local context and working closely with local staff on the intervention development will help make the intervention relevant, acceptable, and scalable. Further, our searches were focused on the three most common mental health problems from our samples, and thus did not include other psychopathology such as trauma and substance use. At this time, youth presenting with these problems would not be able to access school-based services from our program. Moreover, despite high rates of attention problems reported in our sample, the evidence for behavioral intervention for this age group is limited. ADHD is often managed with medication in Western countries. In India, the use of medication for psychosocial problem is limited, largely due to the stigma surrounding the use of psychiatric medication [29]. In addition, academic stress – which is common in India – can manifest as a concentration problem, further reinforcing the stress and worry cycle [30]. Anecdotally, the local clinical team in India indicated that attention problems were largely due to underlying emotional issues, parental pressure and limited resources- and were not persistent in other areas of the adolescents’ lives. Contextual challenges in schools such as crowded classrooms, overburdened teachers, lack of recreational equipment may further exacerbate concentration difficulties. Further research is needed to better understand how academic stress and lack of concentration impacts youth in India, and how this priority area does not align with diagnostic categories. Finally, our help-seeking sample was relatively small (*n* = 78) and the base rate of psychopathology in our epidemiological sample was also low (2.6%, n = 54 of 2,048). This may not be representative of the national averages, which hover around 5% to 7% [31]. Nevertheless, these samples offered a glimpse of the most common mental health problems among Indian adolescents.

## Conclusion

By leveraging what we know works based on the research evidence, and taking into account the local epidemiology, and fit with the context, we expect that this intervention will be acceptable to providers in our collaborating schools, and scalable to other schools in India. The intervention is currently undergoing a clinical case series, which will inform future adjustments to the current iteration of the intervention, based on feedback from providers and adolescents. Following the case series and an anticipated additional round of revisions, the intervention will be tested in a RCT as part of the larger stepped care model. We hope this thoughtful and thorough process can be used as an example for future expansion of evidence-based mental health treatments in other global mental health settings.

## Data Availability

The data that support the findings of this study is available from the corresponding author upon reasonable request.

## Paper context

By leveraging the global evidence base and taking into account the local epidemiology, we were able to develop an intervention that is both evidence-informed and fits with the local context. As such, the intervention has potential to be acceptable to providers and scalable to other schools in India. This process can be used as an example for future development of evidence-based treatments in other global settings.
